# Timeliness of Care-Seeking From Village Health Workers for Children Under Five Years During the COVID-19 Lockdown in Southwestern Uganda

**DOI:** 10.7759/cureus.110837

**Published:** 2026-06-14

**Authors:** Edgar M Mulogo, Peter Chris Kawungezi, Angela Tushabe, Moses Ntaro, Michael Matte

**Affiliations:** 1 Department of Community Health, Mbarara University of Science and Technology, Mbarara, UGA

**Keywords:** child health, community health, covid-19 lockdown, health service access, integrated community case management (iccm), rural health services, timeliness of care-seeking behavior, uganda, under-five children, village health workers

## Abstract

Background

In Uganda, village health workers (VHWs) use the integrated community case management (iCCM) strategy to treat malaria, pneumonia, and diarrhea in the community. It is very important for children to get treatment within 24 hours of getting sick to lower their risk of death and illness. In March 2020, Uganda imposed nationwide COVID-19 lockdown measures, encompassing transport restrictions and curfews, potentially impacting healthcare accessibility. This study evaluated the impact of the lockdown on the timeliness of treatment-seeking from VHWs for children under five years in southwestern Uganda.

Methods

We conducted a retrospective review of VHW patient registers from 22 villages in Bugoye sub-county, Kasese district, for the time between December 1, 2019, and May 31, 2020. The time before the lockdown was from December 1, 2019, to March 22, 2020, and the time during the lockdown was from March 23 to May 31, 2020. There were 4,024 child records in total, and 3,822 of them were used in regression models because they had all the information needed. Timeliness was defined as seeking care within 24 hours of the onset of illness. We used logistic regression to find crude and adjusted odds ratios (ORs) with 95% confidence intervals (CIs).

Results

Overall, a total of 2,428 out of 3,822 (63.5%) children sought care within 24 hours. In the pre-lockdown period, 823 out of 1,330 people (61.9%) sought care on time. During the lockdown, 1,605 out of 2,492 people (64.4%) (p = 0.131) sought care on time. In multivariable logistic regression, the lockdown period was not significantly associated with timely care-seeking (adjusted OR (aOR) = 1.13, 95% CI: 0.99-1.30; p = 0.079). Fever was independently correlated with increased likelihood of timely care-seeking (aOR = 1.18, 95% CI: 1.02-1.37; p = 0.023), and increasing age in months was similarly associated with slightly higher odds (aOR = 1.01 per month increase, 95% CI: 1.00-1.01; p = 0.024). There was no significant association between sex, rapid breathing, diarrhea, and danger signs and timeliness.

Conclusion

The nationwide COVID-19 lockdown period was associated with a modest but non-significant increase in timely care-seeking. These results indicate that community-based health services delivered by VHWs remained accessible and robust despite mobility restrictions. Improving VHW programs could help make sure that important child health services are still available during future public health emergencies.

## Introduction

Timely access to suitable treatment for prevalent pediatric illnesses is fundamental to global child survival initiatives. Malaria, pneumonia, and diarrhea remain significant contributors to morbidity and mortality in children under five years, especially in sub-Saharan Africa [1-3]. Delays in obtaining care, particularly after 24 hours from the onset of symptoms, correlate with heightened disease severity, complications, and mortality [4].

In addressing these challenges, the World Health Organization and United Nations Children's Fund implemented the integrated community case management (iCCM) strategy, which enables community health workers to diagnose and treat uncomplicated cases of malaria, pneumonia, and diarrhea at the community level. This methodology has been extensively implemented in Uganda, where village health workers (VHWs), or village health teams, are essential in providing primary healthcare services in underserved rural areas. The iCCM strategy prioritizes timely treatment within 24 hours of illness onset as a critical performance metric for enhancing child survival rates [5-7].

Data from Uganda and other resource-limited environments indicate that community-based health interventions markedly diminish obstacles to healthcare access, such as distance, expense, and delays linked to facility-based care [8,9]. Evidence from Uganda and other rural settings in sub-Saharan Africa indicates that community-based healthcare delivery through VHWs and community health workers can substantially improve access to timely treatment for childhood illnesses. Studies have shown that proximity of community health workers to households reduces geographical and financial barriers to care and promotes earlier treatment-seeking for malaria, pneumonia, and diarrhea [8,9]. In rural southwestern Uganda, Mulogo et al. reported that approximately two-thirds of children under five years sought treatment from VHWs within 24 hours of illness onset, demonstrating the important role of community-based services in facilitating prompt care-seeking [8]. Care-seeking behavior is influenced by various factors, including caregivers' perceptions of illness severity, sociocultural norms, and the accessibility of health systems [10]. It has been reported in other sub-Saharan African settings, where caregivers were more likely to seek timely treatment when community health workers were readily accessible within their communities [9-11]. Symptoms such as fever and respiratory distress are frequently regarded as more critical, leading to prompt medical attention, whereas conditions like diarrhea may be undervalued, causing deferred treatment [11].

The onset of the COVID-19 pandemic in late 2019 and its ensuing worldwide dissemination prompted unparalleled public health measures. After the confirmation of Uganda's initial COVID-19 case in March 2020, the government instituted rigorous nationwide control measures, such as the suspension of public transport, closure of educational institutions, movement restrictions, and the enforcement of curfews [12]. Although these interventions were vital in curtailing transmission, they also risked obstructing access to essential health services.

Prior epidemics, including the Ebola virus disease outbreak in West Africa, illustrated that disruptions in health systems during public health emergencies can result in decreased healthcare utilization and heightened indirect mortality from otherwise manageable conditions [13,14]. Preliminary evidence from the COVID-19 pandemic indicated declines in outpatient visits, immunization rates, and the utilization of maternal and child health services in numerous low- and middle-income countries [15].

In Uganda, apprehensions were expressed that lockdown measures might obstruct prompt access to healthcare, especially for rural populations reliant on transportation to reach medical facilities [16]. Nevertheless, community-based health delivery platforms, such as VHWs, may have offered a vital safeguard against these disruptions due to their proximity to households and integration within local communities. Nonetheless, there is scant empirical evidence regarding the effectiveness of community health systems in providing timely care during periods of restricted mobility. This study aimed to assess whether the nationwide COVID-19 lockdown affected the timeliness of care-seeking from VHWs among children under five years in southwestern Uganda.

This work was previously presented as a conference abstract at the 2nd International Conference on Public Health in Africa (CPHIA2022) on December 14th 2022. The conference abstract reported preliminary findings from the study. The current manuscript presents a more comprehensive analysis of the data, including multivariable logistic regression modeling, additional methodological details, expanded results, and a more detailed interpretation of the findings than were included in the original abstract.

## Materials and methods

Design and setting

A retrospective analytical study using routinely collected program data extracted from VHW patient registers was conducted in Bugoye sub-county, Kasese district, southwestern Uganda. The setting and data source align with previous research in the same area, which established the feasibility and reliability of utilizing VHW registers to evaluate the timeliness of care-seeking for prevalent childhood illnesses within the iCCM framework [8,9]. Under iCCM, VHWs are instructed to evaluate, categorize, and manage malaria, pneumonia, and diarrhea in children under five years within their communities, thus enhancing access to prompt care in rural and inaccessible regions [5-7].

Data collection

The study population included children under five years old who received care from VHWs. All records of children aged below five years who sought care from VHWs for symptoms consistent with malaria, pneumonia, or diarrhea during the study period (December 1, 2019, to May 31, 2020) were eligible for inclusion. Data were extracted in September 2022 from VHW registers maintained across 22 villages. These registers consistently document essential clinical and demographic variables, such as age, sex, presenting symptoms, date of illness onset, and date of consultation. Records were included if they contained complete information on the date of illness onset and the date of consultation, which were necessary to determine the timeliness of care-seeking. Records with missing or implausible dates (e.g., consultation date preceding illness onset) were excluded from the analysis. In addition, records lacking key covariates required for regression analysis, including age, sex, or presenting symptoms (fever, fast breathing, diarrhea, or danger signs), were excluded from multivariable analysis but retained in descriptive summaries where appropriate. Comparable data collection methodologies have been previously validated in this context to assess the timeliness of care-seeking and the factors influencing prompt treatment [8]. A total of 4,024 child records were identified and incorporated into the descriptive analysis.

To ensure data quality, individuals involved in data extraction were trained on the study objectives, variables of interest, and standardized data abstraction procedures prior to the commencement of data collection. A standardized data extraction form was used to ensure consistency in capturing information from VHW registers. During the extraction process, a random sample of records was independently verified against the original source registers to assess completeness and accuracy. Following data entry, quality checks were conducted to identify duplicate records, missing values, and implausible dates or entries. Identified discrepancies were reviewed and corrected through reference to the original registers before analysis.

Variables and analysis

The primary exposure variable was the period of care-seeking in relation to the nationwide COVID-19 lockdown. The pre-lockdown period was defined as December 1, 2019, to March 22, 2020, while the lockdown period was defined as March 23, 2020, to May 31, 2020, corresponding to the implementation of national restrictions following the confirmation of the first COVID-19 case in Uganda [12]. The primary outcome variable was timeliness of care-seeking, defined as seeking treatment from a VHW within 24 hours of the onset of illness. This definition is consistent with iCCM guidelines and prior studies in this setting, which have used the same threshold to assess promptness in treatment for malaria, pneumonia, and diarrhea among children under five years [7,8].

Covariates included child sex, age in months, and presenting symptoms at consultation, specifically fever (proxy for malaria), fast breathing (proxy for pneumonia), diarrhea, and the presence of danger signs (including inability to drink or breastfeed, persistent vomiting, convulsions, lethargy/unconsciousness, and inability to eat, consistent with Uganda iCCM guidelines). These variables were selected based on prior evidence demonstrating their influence on caregiver decision-making and timeliness of care-seeking, including findings from earlier work in the same population, which showed that symptom type and perceived severity significantly affect prompt care-seeking behavior [8-11]. Of the 4,024 records identified, 202 records were excluded from regression analyses due to missing information on the outcome variable or key covariates. These records were retained in descriptive summaries where possible. Only records with complete data on the outcome and covariates were included in regression analyses, yielding a final analytical sample of 3,822 observations.

Data were analyzed using STATA version 13 (StataCorp, College Station, TX). Descriptive statistics were used to summarize child characteristics and proportions of timely care-seeking, reported as frequencies and percentages for categorical variables and means with standard deviations for continuous variables. Differences in proportions between pre-lockdown and lockdown periods were assessed using chi-square tests, while differences in means were evaluated using Student’s t-tests. Statistical significance was set at p < 0.05.

To evaluate the association between the COVID-19 lockdown and the timeliness of care-seeking, logistic regression analysis was performed. Crude odds ratios (ORs) with 95% confidence intervals (CIs) were first estimated in bivariate models. A multivariable logistic regression model was then fitted to estimate adjusted odds ratios (aORs) with 95% CIs, controlling for potential confounders identified from prior literature and contextual evidence [8-11]. These included age, sex, fever, fast breathing, diarrhea, and danger signs. Logistic regression was selected because the outcome variable (timely care-seeking within 24 hours) was binary. Variables included in the multivariable logistic regression model were selected a priori based on evidence from previous studies, biological plausibility, and their established relevance to care-seeking behavior among caregivers of children under five years. Variables considered important in the conceptual framework for timely care-seeking included study period (pre-lockdown versus lockdown), child age, sex, fever, fast breathing, diarrhea, and danger signs. Given their potential role as confounders, these variables were retained in the multivariable analysis. Model assumptions and fit were assessed prior to interpreting the final multivariable logistic regression model. Multicollinearity among independent variables was evaluated using variance inflation factors (VIFs), and no evidence of problematic multicollinearity was identified. The goodness-of-fit of the final model was assessed using the Hosmer-Lemeshow test. Results indicated adequate model fit, and no major violations of logistic regression assumptions were detected. The final model was therefore considered appropriate for estimating the association between the COVID-19 lockdown period and timely care-seeking while controlling for potential confounding variables. All statistical tests were two-sided.

## Results

A total of 4,024 records of children under five years who sought care from VHWs between December 1, 2019, and May 31, 2020, were identified from the routine registers and included in the descriptive analysis. Of these, 202 records were excluded from the multivariable analyses because of missing information on the outcome variable or key covariates required for regression modeling, resulting in a final analytical sample of 3,822 observations.

Overall, 2,428 of the 3,822 children sought care within 24 hours of illness onset, corresponding to a timely care-seeking prevalence of 63.5% (95% CI: 62.0%-65.0%). During the pre-lockdown period, 823 of 1,330 children sought timely care, representing 61.9% (95% CI: 59.3%-64.5%), compared with 1,605 of 2,492 children during the lockdown period, corresponding to 64.4% (95% CI: 62.5%-66.3%). The difference in timely care-seeking between the two periods was not statistically significant (p = 0.131).

Characteristics of the children

Table 1 shows the characteristics of the children who were part of the analysis, broken down by study period. There were 3,822 records with full data. Of those, 1,330 (34.8%) were from before the lockdown, and 2,492 (65.2%) were from during the lockdown. We looked at the mean age of the children in both time periods and the distribution of important clinical characteristics, such as presenting symptoms and danger signs.

**Table 1:** Characteristics of children by study period (N = 3,822). Values are presented as numbers (percentages) for categorical variables and means (standard deviation) for continuous variables. P-values were obtained using chi-square tests for categorical variables and Student’s t-tests for continuous variables.

Characteristic	Pre-lockdown (n = 1,330), Value	Lockdown (n = 2,492), Value	p-value
Age (months), Mean (SD)	23.1 (+15.8)	21.9 (+15.4)	0.024
Gender - Female	699 (52.6%)	1307 (52.4%)	0.976
Gender - Male	631 (47.4%)	1185 (47.6%)	
Fever - No	578 (43.5%)	1229 (49.3%)	0.001
Fever - Yes	752 (56.5%)	1263 (50.7%)	
Fast breathing - No	707 (53.2%)	1147 (46.0%)	<0.001
Fast breathing - Yes	623 (46.8%)	1345 (54.0%)	
Diarrhea - No	926 (69.6%)	1744 (70.0%)	0.846
Diarrhea - Yes	404 (30.4%)	748 (30.0%)	
Danger sign - No	1317 (99.0%)	2456 (98.6%)	0.284
Danger sign - Yes	13 (1.0%)	36 (1.4%)	

As shown in Table 1, children seen during the lockdown period were younger than those seen during the pre-lockdown period (mean age = 21.9 months vs. 23.1 months, p = 0.024). The proportion of female children was similar in both periods (52.6% in pre-lockdown vs. 52.4% in lockdown, p = 0.976). Fever was reported in 56.5% (752/1,330) of children during the pre-lockdown period compared to 50.7% (1,263/2,492) during the lockdown period (p = 0.001). Fast breathing was reported in 46.8% (623/1,330) of children in the pre-lockdown period and 54.0% (1,345/2,492) during the lockdown period (p < 0.001). The proportion of children presenting with diarrhea was similar between the two periods (30.4% vs. 30.0%, p = 0.846). Danger signs were recorded in 1.0% (13/1,330) of children during the pre-lockdown period and 1.4% (36/2,492) during the lockdown period (p = 0.284).

Timely care-seeking within 24 hours

The distribution of timely care-seeking within 24 hours of illness onset by study period and child characteristics is presented in Table 2. Timely care-seeking was compared across demographic and clinical variables to assess differences in proportions.

**Table 2:** Timely care-seeking within 24 hours of illness onset among children under five years by study period and selected characteristics in Bugoye sub-county, Kasese district, Uganda (N = 3,822). Timely care-seeking was defined as seeking treatment from a village health worker (VHW) within 24 hours of illness onset.

Characteristic	n	Timely seeking care, n (%)	95% CI	p-value
Study period - Lockdown	2492	1605 (64.4)	62.5-66.3	0.131
Study period - Pre-lockdown	1330	823 (61.9)	59.3-64.5	
Gender - Female	2006	1270 (63.3)	61.2-65.4	0.796
Gender - Male	1816	1158 (63.8)	61.6-66.0	
Fever - No	1807	1107 (61.3)	59.0-63.5	0.007
Fever - Yes	2015	1321 (65.6)	63.5-67.7	
Fast breathing - No	1854	1180 (63.6)	61.4-65.8	0.908
Fast breathing - Yes	1968	1248 (63.4)	61.3-65.5	
Diarrhea - No	2670	1717 (64.3)	62.5-66.1	0.137
Diarrhea - Yes	1152	711 (61.7)	58.9-64.5	
Danger sign - No	3773	2401 (63.6)	62.1-65.1	0.278
Danger sign - Yes	49	27 (55.1)	41.2-69.0	
Overall	3822	2428 (63.5)	62.0-65.0	

Overall, 63.5% (95% CI: 62.0-65.0) of children sought care from a VHW within 24 hours of illness onset. Timely care-seeking was 61.9% (95% CI: 59.3-64.5) during the pre-lockdown period and 64.4% (95% CI: 62.5-66.3) during the lockdown period (p = 0.131). Children presenting with fever had a higher proportion of timely care-seeking (65.6%, 95% CI: 63.5-67.7) than those without fever (61.3%, 95% CI: 59.0-63.5; p = 0.007). No statistically significant differences in timely care-seeking were observed according to sex, fast breathing, diarrhea, or danger signs.

Factors associated with timely care-seeking within 24 hours

Logistic regression analysis was conducted to assess factors associated with timely care-seeking within 24 hours of illness onset. Crude and adjusted odds ratios for the association between study period, child characteristics, and timely care-seeking are presented in Table 3.

**Table 3:** Logistic regression analysis of factors associated with timely care-seeking within 24 hours (N = 3,822). Crude odds ratios (ORs) and adjusted odds ratios (aORs) with 95% confidence intervals (CIs) are presented. Statistical significance was set at p < 0.05.

Variable	Crude OR (95% CI)	p-value	Adjusted OR (95% CI)	p-value
Lockdown period (vs. pre-lockdown)	1.11 (0.97-1.28)	0.122	1.13 (0.99-1.30)	0.079
Female sex (vs. male)	0.98 (0.86-1.12)	0.77	0.97 (0.85-1.11)	0.65
Age in months	1.01 (1.00-1.01)	0.005	1.01 (1.00-1.01)	0.024
Fever (yes vs. no)	1.20 (1.05-1.37)	0.006	1.18 (1.02-1.37)	0.023
Fast breathing (yes vs. no)	0.99 (0.87-1.13)	0.882	1.04 (0.89-1.20)	0.636
Diarrhea (yes vs. no)	0.89 (0.78-1.03)	0.127	0.97 (0.82-1.13)	0.674
Danger sign (yes vs. no)	0.70 (0.40-1.24)	0.22	0.70 (0.40-1.25)	0.229

As shown in Table 3, the odds of timely care-seeking during the lockdown period were not significantly different from the pre-lockdown period in both crude analysis (OR = 1.11, 95% CI: 0.97-1.28; p = 0.122) and multivariable analysis (aOR = 1.13, 95% CI: 0.99-1.30; p = 0.079). Female sex was not significantly associated with timely care-seeking in crude (OR = 0.98, 95% CI: 0.86-1.12; p = 0.770) or adjusted analysis (aOR = 0.97, 95% CI: 0.85-1.11; p = 0.650). Increasing age in months was associated with timely care-seeking in crude analysis (OR = 1.01, 95% CI: 1.00-1.01; p = 0.005) and remained significant after adjustment (aOR = 1.01, 95% CI: 1.00-1.01; p = 0.024).

Fever was associated with higher odds of timely care-seeking in crude analysis (OR = 1.20, 95% CI: 1.05-1.37; p = 0.006) and in adjusted analysis (aOR = 1.18, 95% CI: 1.02-1.37; p = 0.023). Fast breathing was not significantly associated with timely care-seeking in crude (OR = 0.99, 95% CI: 0.87-1.13; p = 0.882) or adjusted analysis (aOR = 1.04, 95% CI: 0.89-1.20; p = 0.636).

Diarrhea was not significantly associated with timely care-seeking in crude (OR = 0.89, 95% CI: 0.78-1.03; p = 0.127) or adjusted analysis (aOR = 0.97, 95% CI: 0.82-1.13; p = 0.674). The presence of danger signs was also not significantly associated with timely care-seeking in crude (OR = 0.70, 95% CI: 0.40-1.24; p = 0.220) or adjusted analysis (aOR = 0.70, 95% CI: 0.40-1.25; p = 0.229).

## Discussion

This study assessed the association between the nationwide COVID-19 lockdown and the timeliness of care-seeking from VHWs among children under five years in southwestern Uganda. Overall, 63.5% of children sought care within 24 hours of illness onset. The proportion of timely care-seeking was slightly higher during the lockdown period than during the pre-lockdown period; however, this difference was not statistically significant. In multivariable logistic regression analysis, the lockdown period was not significantly associated with timely care-seeking after adjusting for child age, sex, fever, fast breathing, diarrhea, and danger signs. Fever and increasing age in months were independently associated with higher odds of timely care-seeking, while sex, fast breathing, diarrhea, and danger signs were not significantly associated with the outcome. These findings suggest that timely access to care through VHWs was largely maintained during the COVID-19 lockdown.

The findings of this study are consistent with previous evidence from Uganda and other rural sub-Saharan African settings that demonstrate the contribution of community health workers to timely access to treatment for childhood illness. In rural southwestern Uganda, Mulogo et al. found that a substantial proportion of caregivers sought treatment from VHWs within 24 hours of illness onset, highlighting the effectiveness of the integrated community case management strategy in promoting prompt care-seeking [8]. Studies conducted elsewhere in sub-Saharan Africa have similarly reported that community health workers improve access to treatment by reducing travel distance, minimizing out-of-pocket costs, and increasing the availability of healthcare services within communities [9-11]. The similarity in timely care-seeking observed before and during the lockdown in the present study extends this body of evidence by suggesting that community-based healthcare delivery may continue to facilitate access to essential child health services even during periods of restricted mobility. One possible explanation for the absence of a significant decline in timely care-seeking during the COVID-19 lockdown is that VHWs are located within the communities they serve and may therefore have remained more accessible than facility-based services during periods of restricted movement. Although accessibility and proximity were not directly measured in this study, previous research has shown that community-based health workers can reduce geographical barriers to healthcare and improve timely access to treatment for common childhood illnesses [8-11]. The findings of the present study are consistent with this possibility, although further research is needed to directly assess the mechanisms through which community-based services maintained access to care during the lockdown period.

Timely healthcare-seeking is a vital factor in child survival, especially concerning malaria, pneumonia, and diarrhea, which continue to be predominant causes of mortality in children under five years worldwide [1-3]. Prolonged delays in obtaining treatment correlate with a heightened risk of complications and mortality [4]. The iCCM strategy was developed to mitigate delays by providing essential treatment nearer to households via trained community health workers [5-7]. This study's findings empirically validate the efficacy of this strategy in ensuring timely access to care during a public health emergency.

The lack of a notable decrease in timely care-seeking during the COVID-19 lockdown contradicts apprehensions that movement restrictions would severely hinder access to vital health services [12]. Data from prior epidemics, such as the Ebola outbreak in West Africa, indicated significant declines in healthcare utilization and heightened indirect mortality resulting from interruptions in routine services [13,14]. Early projections during the COVID-19 pandemic indicated that disruptions in essential health services could significantly elevate maternal and child mortality rates in low- and middle-income countries [15], prompting national guidance in Uganda to maintain essential health services during the pandemic [16]. The study's findings indicate that decentralized, community-based healthcare delivery models, such as services led by VHWs, may alleviate these disruptions.

The resilience demonstrated in this study is likely due to the closeness of VHWs to households in their communities. In contrast to facility-based services that necessitate transportation, VHWs deliver care within proximity, thereby diminishing dependence on mobility and alleviating obstacles during lockdown scenarios. This aligns with prior evidence indicating that community health workers enhance access to care by mitigating geographical and financial obstacles [8,9]. Moreover, previous research in the same context demonstrated that timely care-seeking from VHWs is viable and affected by accessibility and caregivers' perceptions of illness severity [8].

This study found that fever was independently linked to increased likelihood of timely care-seeking, whereas other symptoms like diarrhea and rapid breathing did not show a significant correlation with timeliness. The findings align with prior research suggesting that caregivers are more inclined to pursue immediate medical attention for symptoms deemed severe or life-threatening, such as fever, frequently linked to malaria [10,11]. Conversely, diarrhea may be regarded as less pressing, resulting in postponements in pursuing medical care. The correlation between increasing age in months and marginally elevated probabilities of timely care-seeking may indicate enhanced caregiver experience or improved recognition of illness patterns in older children.

The results of this study hold significant implications for health system resilience and emergency preparedness. Enhancing community-based health systems, especially VHW programs, could be a vital approach to maintaining essential health services during emergencies. The capacity of VHWs to sustain service delivery during the COVID-19 lockdown underscores their function as a pivotal element of the primary healthcare system in rural areas. Incorporating VHWs into national emergency response frameworks and guaranteeing a steady supply of essential medicines and oversight may augment their efficacy in future public health crises.

While the findings suggest that VHW programs can contribute to maintaining access to essential child health services during public health emergencies, sustaining these programs may be associated with several operational challenges. Community-based health workers frequently operate in resource-constrained environments and may experience interruptions in the supply of essential medicines and diagnostic commodities during emergencies. In addition, effective program implementation requires ongoing supervision, refresher training, and logistical support, which may be difficult to maintain during periods of restricted movement or competing health system priorities. Transport constraints, communication challenges, and limited funding may further affect the ability of VHWs to provide consistent services. Therefore, efforts to strengthen community-based health systems should be accompanied by adequate investments in supply chain management, supervision, training, and sustainable financing to ensure continuity of care during future public health emergencies.

This research possesses numerous strengths. It employed an extensive dataset of more than 4,000 child records obtained from standard VHW registers, offering empirical evidence of care-seeking behavior. The application of descriptive and multivariable regression analyses facilitated the adjustment of potential confounders and enabled a more rigorous evaluation of associations. The study further develops previously suggested methodologies employed in the same context, thereby augmenting the comparability and credibility of the findings [8].

Study limitations

Several limitations of this study should be considered when interpreting the findings. First, the retrospective analysis relied on routinely collected VHW records, which may be subject to incomplete documentation and recording errors. Although data quality assurance procedures were implemented, approximately 5% of records were excluded from regression analyses because of missing information on the outcome or key covariates. Second, the study lacked information on caregiver- and household-level factors, such as socioeconomic status, education, and perceptions of illness severity, which may influence care-seeking behavior. Third, while the findings suggest that VHW services remained accessible during the COVID-19 lockdown, accessibility and health system resilience were not directly measured in this study. Rather, these interpretations were inferred from the observed maintenance of timely care-seeking during the lockdown period and should therefore be interpreted cautiously. Future studies incorporating direct measures of service accessibility, health system functionality, and caregiver experiences would provide a more comprehensive understanding of the mechanisms underlying continuity of community-based care during public health emergencies. Finally, because the study was conducted in a rural setting in southwestern Uganda, the findings may not be generalizable to urban areas or settings with different health system contexts.

## Conclusions

The national COVID-19 lockdown period showed a modest but non-significant increase in timely care-seeking in southwestern Uganda. The findings indicate that community-based health services provided by VHWs remained accessible and resilient during mobility restrictions. Strengthening and maintaining VHW programs could improve the continuity of vital child health services during forthcoming public health crises.
